# Modulation of antioxidant systems and photosynthetic machinery by foliar-applied ZnO nanoparticles in cadmium-stressed mung bean (*Vigna radiata* L.)

**DOI:** 10.1186/s12870-026-08452-7

**Published:** 2026-03-11

**Authors:** Eram Shahzadi, Muhammad Humza, Muhammad Shahid, Sajad Hussain, Ulkar Ibrahimova, Hamideh Ghaffari, Yang Liu, Xinghong Yang, Marian Brestic

**Affiliations:** 1 College of Life Science, State Key Laboratory of Crop Biology, Shandong Key Laboratory of Crop Biology, Shandong Agricultural University, Tai’an, 271018 China; 2 Institute of Plant and Environmental Sciences, Faculty of Agrobiology and Food Resources, Slovak University of Agriculture, Trieda A. Hlinku 2, Nitra, 949 76 Slovakia; 3 State Key Laboratory of Wheat Improvement, Shandong Provincial Key Laboratory of Agricultural Microbiology, College of Plant Protection, Shandong Agricultural University, Tai’an, 271018 China; 4 Plant Breeding and Genetics Division, Nuclear Institute of Agriculture and Biology (NIAB), Faisalabad, 38000 Pakistan; 5 National Research Center of Intercropping, The Islamia University of Bahawalpur, Bahawalpur, 63100 Pakistan; 6 Institute of Molecular Biology Public Legal Entity, Ministry of Science and Education of the Republic of Azerbaijan, 11 Izzat Nabiyev, Baku, AZ 1073 Azerbaijan; 7 Nanotechnology and Biochemical Technology (NBT) center, Azerbaijan State University of Economics (UNEC), Baku, AZ 1001 Azerbaijan; 8 AgroBioTech Research Center, Slovak University of Agriculture, A. Hlinku 2, Nitra, Slovakia

**Keywords:** Cadmium, Zinc oxide nanoparticles, Chlorophyll, Stress, Yield

## Abstract

Agricultural soil contamination with cadmium (Cd) presents a significant threat to plant growth and food security. Nanotechnology offers innovative approaches to mitigate the adverse effects of heavy metal stress on plants. This study investigated the impact of foliar-applied zinc oxide nanoparticles (ZnO-NPs) on mung bean (*Vigna radiata* L.) plants subjected to Cd stress. To induce Cd stress in mung bean plants, varying concentrations of cadmium were applied: control (no Cd), 50 µM, and 100 µM, using cadmium chloride (CdCl_2_) as the source. ZnO-NPs were administered via foliar spraying at different concentrations (40 and 80 mg/L). Mung bean varieties, NIAB Mung 2011 (NM-2011) and NIAB Mung 2021 (NM-2021), were treated with ZnO-NPs under Cd stress conditions. Our results demonstrated that ZnO-NPs significantly mitigated the detrimental effects of Cd stress on mung bean cultivars. The application of ZnO-NPs (80 mg/L) notably enhanced plant growth under both 50 µM and 100 µM Cd stress levels. The findings revealed a dose-dependent effect, with optimal ZnO-NPs concentrations promoting superior shoot (17%) and root growth (20%); increased chlorophyll content (total chlorophyll 39%, chlorophyll *a* 38%, chlorophyll *b* 41%, carotenoids 40%), indicating improved photosynthetic performance. ZnO-NPs also elevated the activity of antioxidant enzymes such as superoxide dismutase (SOD) (32%), catalase (CAT) (19%), ascorbate peroxidase (APX) (35%) and peroxidase (POD) (26%), and reduced oxidative stress induced by Cd toxicity also along with decreased lipid peroxidation (MDA 35%, H_2_O_2_ 63%). These findings underscore the potential of nanotechnology-based strategies to enhance plant resilience to heavy metal stress, which is crucial for sustainable agricultural practices. Further investigation is warranted to elucidate the underlying mechanisms and assess the long-term impacts of ZnO-NPs on plant growth and cadmium stress alleviation.

**Supplementary Information:**

The online version contains supplementary material available at 10.1186/s12870-026-08452-7.

## Introduction

Abiotic stress is the major aspect impacting agricultural development and production globally [1]. Plants are continually exposed to adverse environmental conditions such as salt, heat, drought, and heavy metal pollution. Heavy metals have been shown to lower agricultural yield by interfering with plant physio-biochemical processes [2]. Cadmium (Cd), a highly toxic heavy metal ranked seventh among the top twenty hazardous substances, is commonly introduced into arable soils through industrial and agricultural activities [3, 4].

Although Cd is highly mobile in the phloem, it could accumulate in all parts of a plant, causing stunted growth, altering the ultrastructure of chloroplasts, inhibiting photosynthesis, inactivating enzymes of CO_2_ fixation, inducing lipid peroxidation, and interfering with nitrogen (N) metabolism as well as antioxidant machinery [5]. It may also impede the function of several enzyme families, including those involved in the Calvin cycle, glucose metabolism, and the metabolism of phosphorus [6].

Plant roots have been identified as the initial contact site for hazardous metals [7]. Cd ion buildup in tissues influences water absorption from the soil, which reduces water uptake [8]. Its presence inside the soil may influence root absorption as well as the distribution of nutrients and movement, including Ca, P, Mg, and K [9]. It causes cell death, lipid peroxidation, and enzyme inhibition by encouraging the development of reactive oxygen species (ROS) in the cells of plants [10, 11].

Plants employ three mechanisms to deal with Cd toxicity in plants is avoidance, tolerance and adaptation [12]. Limiting Cd accumulation in the plant is part of the avoidance strategy [13]. The resistance mechanism consists of Cd storage and accumulation through peptide binding, proteins, and amino acids [14]. Plants’ responses to Cd stress are connected to stress signaling systems, including jasmonic acid, humic acid, methyl jasmonate, salicylic acid, nitric oxide, and ethylene [15]. Plants utilize a ROS detoxifying antioxidant defense system that comprises non-enzymatic (glutathione, GSH; proline, glycine betaine, ascorbic acid, flavonoids, phenolics) and enzymatic antioxidants to counteract the inhibitory effects of ROS [16].

Nanoparticles (NPs) are now being used as a novel method to mitigate Cd stress while functioning as nano-fertilizers [17]. As a result, the involvement of nanoparticles with plant growth, especially under stress conditions, has been clearly demonstrated, with NPs being utilized to transport proteins, specific chemicals, and nucleotides to specific targets [18]. Various factors influence the Zn availability, including pH, physicochemical characteristics, and crop tolerance level [3, 19]. Zinc oxide nanoparticles (ZnO-NPs) are found to be effective in reducing the impact of cadmium, which provides a potential pathway to enhance agricultural productivity within contaminated environments owing to their high catalytic activity and large surface area [20, 21]. Sun et al. [22] reported that ZnO-NPs provide extensive capability to alleviate abiotic stress by enhancing salt tolerance via reprogramming of carbon, nitrogen, and other metabolites. Zou et al. [23] concluded that ZnO-NPs alleviate cadmium toxicity in maize grown in soil. ZnO-NPs play a critical role in antagonistic interface with cadmium, which depicts their critical significance in mitigation of cadmium accumulation in plants, as zinc reduces the potential of cadmium for uptake by transporters and absorption in plant tissues [21, 24]. Rashid et al. [25] confirmed that application of ZnO nanoparticles at 8µM concentration significantly improved the root growth and shoot growth up to appreciable extent however, the antioxidant enzymes were significantly boosting reducing the impact of Cd toxicity in mungbean cultivars.

Mung bean (*Vigna radiata* L.) is extensively grown and utilized in Asia; however, it is vulnerable to the stress of Cd. It is a short-duration, biannual, warm-season leguminous crop that is extensively produced as a food crop, particularly in underdeveloped nations. There is limited literature on Cd toxicity tolerance in mung bean, and this lack of knowledge has hampered agricultural progress in this crop. The impact of ZnO-NPs during Cd stress has not been adequately investigated. So, there is a need to reduce Cd translocation from soil to aerial parts of plants, as this is becoming an increasingly concerning problem. Therefore, the present study aimed to investigate whether foliar spraying of ZnO-NPs may boost photosynthesis and improve antioxidant profile, and nutrient absorption in mung bean cultivars by restricting Cd uptake, thus boosting growth and production under the toxic heavy metal stress. Moreover, it will find out the optimal dosage of ZnO-NPs in reducing the cadmium levels without posing any toxic impact on the environment, favoring the agenda of one-health and addressing key UN-SDGs.


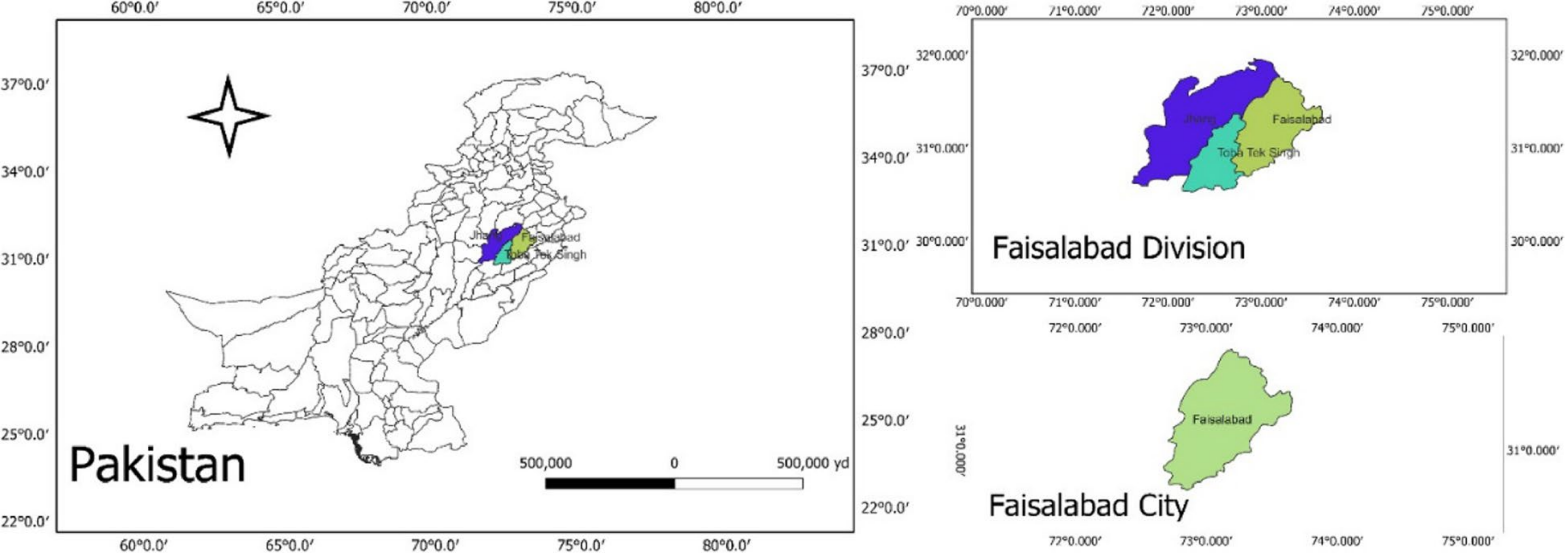


## Materials and methods

### Nanoparticle characterization & application

Sigma-Aldrich provided the ZnO-NPs (average particle size 150 nm), an American-based company (St. Louis, MO, USA). According to the company’s manufactured ZnO NPs, the Assay is approximately. 80% zinc basis having a molecular weight of 81.39 g/mol. X-Ray diffraction spectroscopy revealed 99% purity with average particle size < 50 nm, 10-25m^2^/g surface area, and 5.61 g/m^3^ density. The Zeta Potential of ZnO-NPs in double-distilled water was 27mV with marginal stability. Preparing a suitable amount of NPs at a rate of 100 mg/L was accomplished by dissolving the desired amount of NPs with double-distilled water (DDW) at a volume of 10 mL into a volumetric flask of 100 mL, and then creating the volume by using the DDW. For uniform coverage of the applied solution, 0.05% Tween 20 was added to the above solution [26].

### Experiment setup

This study aimed to evaluateZnO-NPs on cadmium-stressed mung bean (*Vigna radiata* L.) plants. This experiment was carried out in soil and silt-filled pots. Soil is sandy loam with a pH of 7.82 and a content of 0.82% organic matter, 0.042% nitrogen, 6.65 mg/L bioavailable phosphorus, and 160 mg/L potassium. Di-ammonium phosphate (5.50 g) and potassium sulphate (1.82 g) were supplied to each pot to address nutritional demands. Silt and soil (6 kg) were appropriately combined (1:2) in the pots. Varying levels of Cd stress, i.e., control (no Cd), 50 and 100 µM, along with three levels of ZnO-NPs application, such as control, 40 and 80 mg/L [27, 28]. The Cd source was cadmium chloride, fully incorporated in the soil. In each pot, CdCl₂ solution at 200 mL per pot was applied, totaling the concentrations of 50 µM and 1000 µM into the substrate two times, at an interval of two days between applications, in order to allow distribution through irrigation. The seeds of mung bean varieties NM-2011 (NIAB Mung 2011) and NM-2021 (NIAB Mung 2021) were procured from the Seed Storage Bank in the Plant Breeding Division of Nuclear Institute of Agriculture and Biology (NIAB), Faisalabad 38,000, Pakistan, through the proper channel. Seeds were stored at 4 °C for further use. No wild collection is involved in this study. Experimental procedures complied with the regulations of NIAB, Faisalabad, for research on mung bean cultivars. These varieties have different stress tolerance profiles as well, and both are agronomically significant.

Healthy seeds were sterilized in a 5% solution of sodium hypochlorite. Then, 10 seeds were sown at 1 cm in each container. Weeding and thinning were undertaken after seven days of germination to maintain healthy and uniform seedlings. The Cd stress was maintained to ensure consistent stress levels without causing seedling mortality. Foliar spray of NPs was done at two growth stages, viz. vegetative and flowering, where one liter solution of NPs was prepared, and half of the solution was used at every growth stage. Foliar spray was done approximately. 33mL per plant from a conventional hand sprayer with a droplet size of around 100µM. Furthermore, pots were checked regularly, and the irrigation need was met by watering pots based on visual experience. The experiment was designed using a complete randomized design (CRD) with factorial arrangement including three Cd levels (0, 50 and 100 µM), three ZnO-NP concentrations (0, 40 and 80 mg/L), and two cultivars, NM-2011 and NM-2021, with three replicates per treatment. The levels of cadmium and ZnO-NPs were selected based on the previous studies conducted and these levels were significantly effective in reducing the cadmium level without showing any side effects to environment and living beings.

### Growth traits

The root & shoot lengths (cm) were measured using a scale rod. The root and shoot fresh weight (g) were measured using a digital balance (Scale-Tec, India). Following this, samples were oven dried at 72 °C for 06 h, and the dry weight of the root and shoot (g) was recorded on a digital balance (Scale-Tec, India). All attributes were measured in triplicate.

### Pigment and gas exchange

#### Photosynthetic pigments measurement

Arnon’s [29], the method has been employed to determine the amount of chlorophyll and carotenoid concentration. For the chlorophyll estimation, 0.2 g leaf sample was crushed in 80% acetone (5 mL). Filtered the extract using a Whatman # 02 filter paper, and a spectrophotometer was utilized to measure absorption at 645, 663, and 480 nm. (Hitachi U-2910, Tokyo, Japan). All measurements were conducted in triplicate.

#### Measurement of photosynthetic parameters

We determine gas exchange parameters using the second topmost leaf as described by Faizan et al. [30]. The photosynthesis measuring system CI-340 portable infrared gas analyzer (Analytical Development Company, Hoddesdon, USA) was used to record data. Data was subjected to three replicates per treatment.

### Oxidative stress biomarkers

#### Protein estimation

Following the homogenization of the fresh material of the leaf using liquid nitrogen, 10 mL of cooled 50 mM potassium phosphate buffer (PPB) was poured. Then was centrifuged at 12,000×g for 5 min. Then taken the supernatant was then taken and utilized to calculate total soluble protein as described by Bradford [31]. All measurements were conducted in triplicate.

#### Determination of sugar

Anthrone reagent was used to calculate the sugar content [32]. The whole soluble sugar concentration was calculated using an analytical grade glucose standard curve. All measurements were conducted in triplicate.

#### Total phenolics content (TPC)

TPC in seedlings was determined using the Julkenen-Titto method [33]. The absorbance of the resultant substance was determined by using a UV-Visible spectrophotometer (Hitachi U-2910, Tokyo, Japan) at 750 nm. All measurements were conducted in triplicate.

#### Total flavonoid content (TFC)

TFC was estimated using a colorimetric method established by Zhishen et al. [34] making use of catechin reference solutions. The reading was measured at 510 nm against a suitable blank. All measurements were conducted in triplicate.

#### Proline contents estimation

Sulphosalicylic acid (3%) was used to crush the seedlings, then centrifuged at 10000x g for 10 min to estimate free proline concentrations. A test tube was filled with the same volume of the extracted material was then combined with the glacial acetic acid and the ninhydrin reagent. After 1 h of incubation in a boiling water bath at 100 ˚C, ice was applied to stop the process. Toluene was added to the solution, which was thoroughly mixed. From the aqueous phase, aspiration of the chromophore toluene layer was done as well, and its optical density (OD) was noted at 520 nm [31]. All measurements were conducted in triplicate.

#### Glycine betaine determination (GB)

Fresh extracts of leaves were generated and chilled in 2N H_2_SO_4_ to estimate GB using Grieve and Grattan’s methodology [35]. These extracts were subsequently vortexed and kept at 4 °C with an equal quantity of periodate made by adding excess iodine to a solution of potassium iodide. The solution was centrifuged for fifteen minutes at 10,000 rpm at 4 °C, removed the supernatant was removed. The periodide crystals containing the test tube were added with 10 mL of 1, 2 dichloroethane then vortexed, and allowed to stand for 15–20 min until the absorption of the colored solution at 365 nm. All measurements were conducted in triplicate.

#### H_2_O_2_ measurement

In 20% v/v H_2_SO_4_, 3 mL of the leaves extract mixture, and 1 mL of 0.2% titanium sulphate were added. The reaction mixture was then centrifuged for 10 min at 8000 rpm, and the supernatant was used to measure absorbance at 410 nm wavelength. Then 0.28^− 1^ cm^− 1^ extinction coefficient was used to measure the H_2_O_2_ concentration by the Jana and Choudhuri method [36]. All measurements were conducted in triplicate.

#### Lipid peroxidation (MDA)

Lipid peroxidation level in fresh leaves was estimated by assessing Malondialdehyde (MDA), a dissolution product of the membrane lipid’s peroxidation, using Thio barbituric acid (TBA) was measured using the method of Heath and Packer [37]. All measurements were conducted in triplicate.

### Elemental analysis

#### Nutrient contents determination

Plant shoots/roots were washed twice using double-distilled water before immersing in EDTA (20 mM) for 3 s and rinsed twice more using deionized water to remove adsorbed metal. After washing, the plant materials were dried in an oven at 105 °C for 24 h. The dried shoots and roots were digested in HNO_3_: HClO_4_ (7:3 V/V), and wet digestion was used until clean samples were obtained. Each sample was diluted and filtered to 50 mL with double-distilled deionized water. The contents of K, Ca, Mg, and were determined using an Atomic Absorption Spectrophotometer (Hitachi U-2001, Japan), while P contents were determined following the method of Jackson using a Spectrophotometer (Hitachi U-2910, Tokyo, Japan). All measurements were conducted in triplicate.

### Analysis of root exudates

Freeze-dry exudates were mixed with 80% ethanol to determine the amount of organic acids before being injected through a C18 column. HPLC with the Flexer FX-10 UHPLC isocratic pump was utilized to conduct organic acids quantitative analysis in root exudates (PerkinElmer, MA, USA). Used mobile phase used within HPLC has been an acidic solution of acetonitrile with acetonitrile: H_2_SO_4_: acetic acid ratios of 15:4:1 and a pH of 4.9. The samples have been examined for 10 min. at a flow rate of 1.0 ml min^− 1^. The column’s interior temperature was set to 45 °C, and organic acid measurement was performed at 214 nm wavelength [38]. The samples have been mixed following freeze-drying in double-distilled water, as well as the exudates. A pH meter was used to measure the pH. And an LL micro-pH glass electrode (ISTEK Model 4005–08007 Seoul, South Korea). All measurements were conducted in triplicate.

### Osmotic traits

#### Estimation of leaf water potential (Ψ_w_)

To analyze leaf water potential, the fully grown and young second leaf down from the plant’s top was used. Using a pressure chamber of the Scholander type, measurements were recorded from 8 a.m. to 10 a.m. Typically, four leaves per treatment were measured to account for variability within plants, with three biological replicates utilized to ensure statistical reliability. All measurements were conducted in triplicate.

#### Osmotic potential (Ψ_s_)

Osmotic potentials were determined using four leaves from each treatment, with three biological replicates per calculation. To obtain cell sap, leaf samples have been frozen at ‒20 °C, then thawed and crushed with a glass rod. The sap was sucked using a disposable syringe and immediately quantified using an osmometer (Wescor 5500) for Ψ_s_ determination. All measurements were conducted in triplicate.

#### Pressure potential (Ψ_p_)

The difference between Ψ_w_ and Ψ_s_ values was used to compute the pressure potential

$$\text{}\left(\text{}{{\text{}\Psi_\mathrm{p}}}\text{}\right)=\left(\text{}{{\Psi_\mathrm{w}}}\right)-\left(\text{}{{\Psi_\mathrm{s}}}\right)$$

#### Relative water content

The standard method of Mostofa and Fujita [39] was utilized to measure RWC. The fresh weight was determined by removing the second leaf from different mung bean seedlings and weighing it (FW). The leaves were soaked in distilled water for 24 h before being placed in the dark. The leaves were allowed to dry in the open air before being weighed to estimate their turgid weight. After that, to calculate dry weight, the samples were oven dried at 70 °C for two hr. All measurements were conducted in triplicate. The following formula was used to determine RWC:

$$\:\mathrm{RWC}{}{=}\frac{\text{Fresh weight}-\text{Dry weight}}{\text{Turgid weight}-\text{Dry weight}}{\times}\text{}\mathrm{100}$$

### Assessment of cadmium concentration in plant organs grown in soil

Plant samples were taken, dried, and preserved (roots, stems, leaves, and grains). Following that, 0.5 g of each plant was digested after being ground into powder in a 2:1 mixture of HNO_3_: HClO_4_ [40]. The Cd content in plant organs was evaluated by an atomic absorption spectrophotometer (Hitachi U-2001, Japan). All measurements were conducted in triplicate.

### Enzymatic antioxidants

#### SOD, CAT, POD, and APX activities determination

The SOD content was measured with the Giannopolitis and Ries methodology [41]. The POD activity was measured following the method of Chance and Maehly [42]. Hatch and Glasziou’s method [43] was used to determine CAT activity. The functioning of APX was assessed using the Asada and Takahashi method [44]. For the enzyme analysis, leaf samples were taken into consideration, and all measurements were conducted in triplicate.

#### Estimation of reduced glutathione (GR), oxidized glutathione (GSSG), and glutathione disulfide (GSSG)

The Cakmak et al. [45] Methodology was used to monitor GR activity. To demonstrate GR activity, the increase in absorbance was plotted against a known quantity of glutathione reductase.

Griffith’s method [46] was used to determine the levels of oxidized (GSSG) and reduced (GSH) glutathione in mung bean leaves. Briefly, 250 mg of fresh leaf material was completely crushed in 0.1 M HCl (2 mL), including EDTA (1 mM). Remove the supernatant by centrifuging the extract for 15 min at 4 °C at 12,000 rpm. The resulting mixture was composed of 200 L of phosphate buffer (125 M) comprising 6.3 mM EDTA (pH 7.5), 200 µL of extract, 100 µL of DTNB (6.0 mM), and 500 µL NADPH (0.3 mM). At a wavelength of 412 nm, the mixture’s absorbance was measured. All measurements were conducted in triplicate.

### Yield components

#### Days to maturity

The plant was monitored daily to record the days it took from emergence to harvest. Each plot has two plants picked at random and tagged to determine the number of days to maturity. Data was recorded in three replications.

#### Harvest index and seed yield

Total crop biomass and seed production were measured after harvesting and sun drying. The harvest index has been determined as the ratio of seed yield to total biological yield (above ground). Data was recorded in three replications.

#### Statistical tools

ANOVA was performed to assess the collected data using CoStat software version 6.303 and the LSD (*p* < 0.05) test. Moreover, Origin-Pro 2019 was used to create the graphical presentation. Pearson’s correlation and principal component analysis were also calculated using R Studio.

## Results

### Growth and biomass

**Fig. 1:**
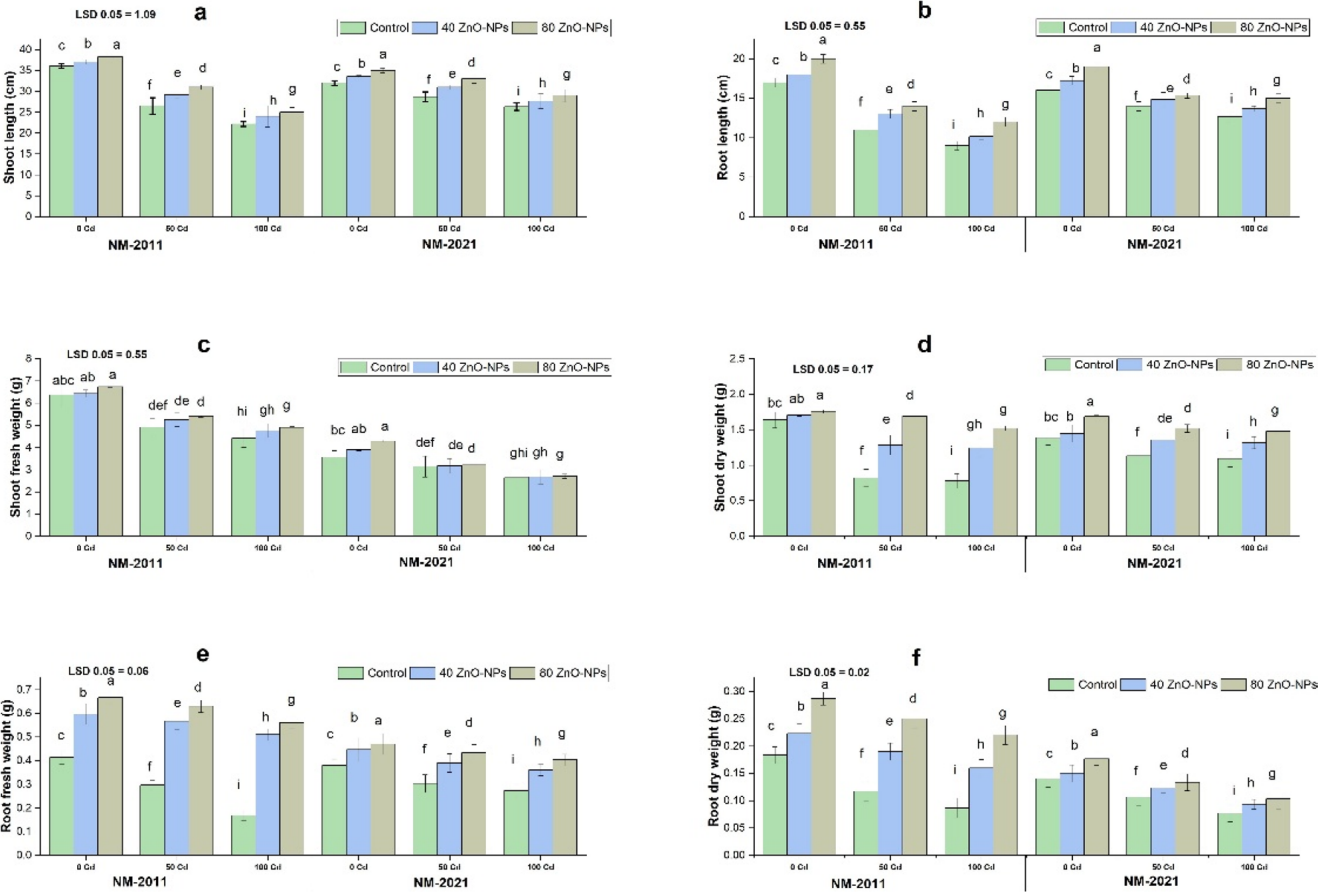
Evaluation of the impact of ZnO-NPs on growth and biomass parameters, including (**a**) shoot length, (**b**) root length, (**c**) shoot fresh weight, (**d**) shoot dry weight, (**e**) root fresh weight, (**f**) root dry weight of two mung bean cultivars viz. NM-2011 and NM-2021 under the cadmium stress.

It is generally known that Cd reduces biomass output greatly and even causes the death of essential plant parts. It retarded mung bean growth in the present study. Growth attributes of both mung bean varieties significantly (*P* < 0.001) reduced at 50 and 100 µM Cd concentration, in contrast with unstressed plants (0 µM Cd). Significant reduction was noted in shoot length, root length, shoot fresh weight, shoot dry weight, root fresh weight, and root dry weight at 100 µM Cd concentration in NM-2011 and NM-2021 varieties as compared with the control. Zinc treatments in various forms are being shown to protect plants from Cd stress and increase plant development. Application of ZnO-NPs (40, 80 mg/L) effectively improved all growth parameters studied under both levels of Cd (50 µM and 100 µM) stress. In the present investigation, we observed that 80 mg/L ZnO-NPs was more effective in reversing the toxic effect of both Cd (50 and 100 µM) stress levels in both varieties (Fig. 1).

### Photosynthesis and gas exchange

**Fig. 2:**
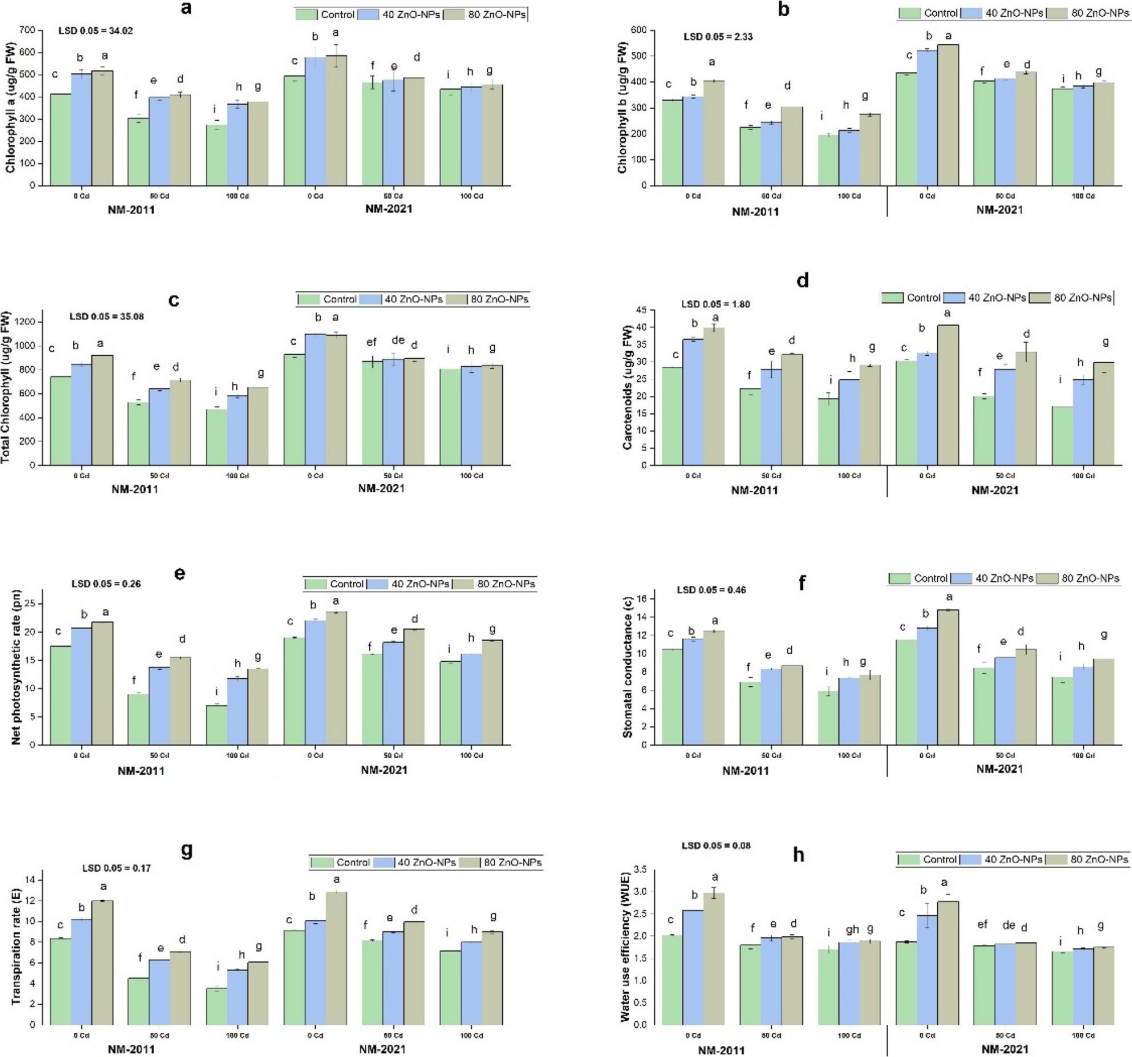
Impact of ZnO-NPs on photosynthetic parameters (**a**) chlorophyll *a* (**b**) chlorophyll *b* (**c**) total chlorophyll (**d**) Carotenoids (**e**) net photosynthetic rate and gaseous exchange parameters including (**f**) stomatal conductance (**g**) transpiration rate (**h**) water use efficiency (WUE) of two mung bean genotypes NM-2011 and NM-2021 under cadmium stress

Photosynthetic pigments were substantially (*P* < 0.005) reduced in both levels of Cd (50 and 100 µM) stress. In the current study, a 100 µM Cd level was more toxic towards photosynthetic pigments. A maximum decrease in chlorophyll *a*, chlorophyll *b*, total chlorophyll, and carotenoid contents was noted at 100 µM Cd concentration in both varieties as compared to the control. Both levels of ZnO-NPs effectively reversed the damage of photosynthetic pigments caused by Cd toxicity. However, we noted that 80 mg/L ZnO-NPs was more effective in relieving Cd toxicity in both varieties under study. The effect of recovery was more prominent in the NM-2021 variety (Fig. 2).

Cd entry into plants via roots can cause toxicity symptoms, which are manifested by alterations in physiological parameters, including gas exchange attributes. The results of this study showed Pn, Gs, E, and WUE contents decreased at Cd (50 and 100 µM) concentration in both varieties, in contrast to the control. However, treatment of ZnO-NPs markedly increased gas exchange characteristics under both levels of Cd (50 µM and 100 µM) stress. In the present investigation, 80 mg/L ZnO-NPs were more effective in reversing the toxic effect of both Cd (50 and 100 µM) stress levels in both varieties (Fig. 2).

### Biochemical indicators

**Fig. 3:**
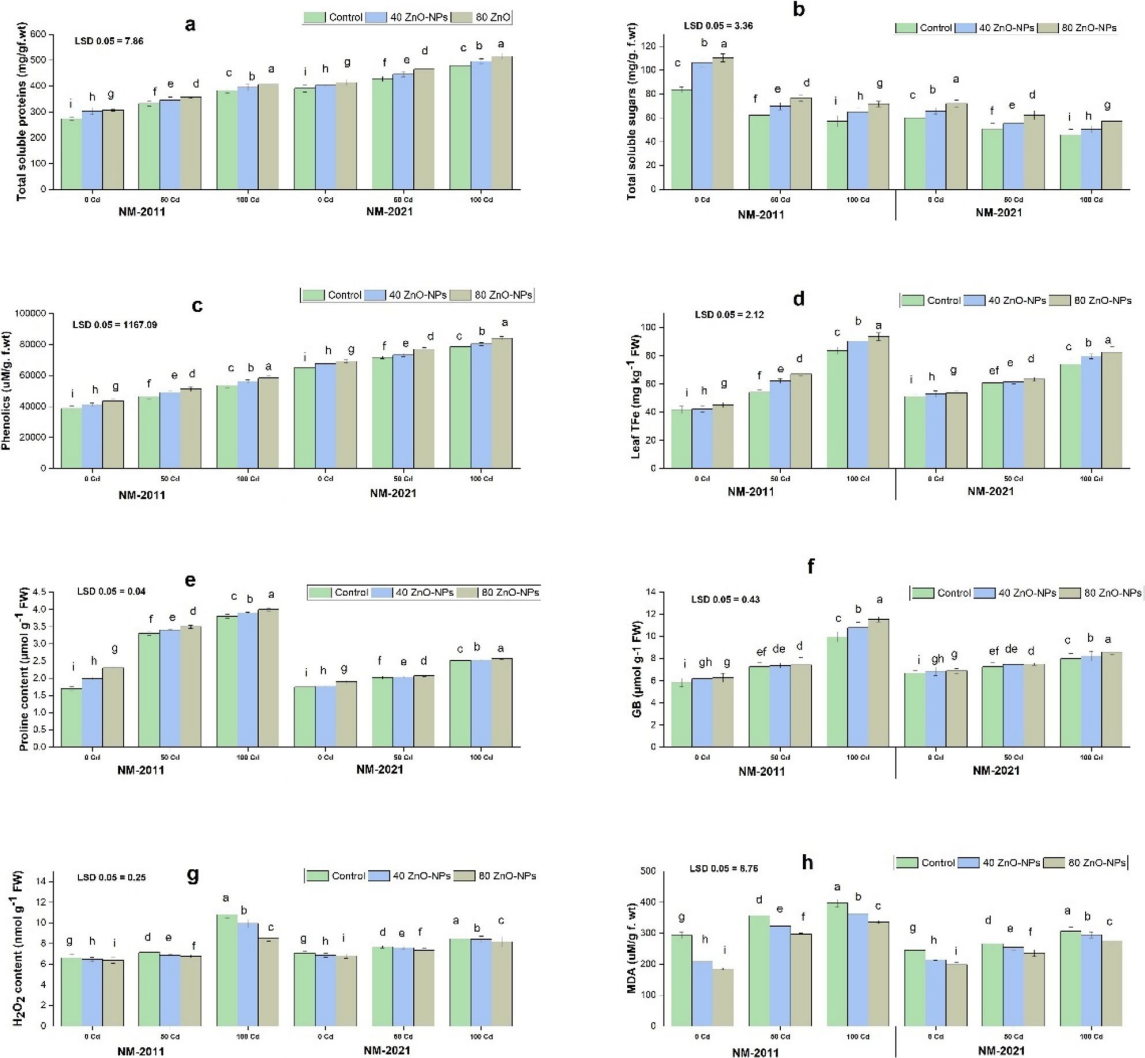
Assessing various concentrations of ZnO-NPs on physiological parameters, including (**a**) total soluble proteins, (**b**) total soluble sugars, (**c**) phenolics, (**d**) total phenolic compounds, (**e**) proline contents, (**f**) glycine betaine, (**g**) hydrogen peroxide, and (**h**) malonaldehyde levels of two mung bean varieties, viz. NM-2011 and NM-2021 under the cadmium stress

Our research showed that the protein content rose in mung bean varieties subjected to Cd stress. The current results indicated that protein levels increased at a Cd concentration of 50 µM in both varieties. However, a more significant increase in protein content was observed at a 100 μm Cd concentration in the NM-2021 variety compared to control plants. Additionally, a substantial increase in protein content was noted with the addition of ZnO-NPs. In response to Cd 100 µM, the presence of ZnO-NPs at 80 mg/L in NM-2021 varieties further elevated the protein content (Fig. 3).

The total soluble sugar content in two varieties of mung beans was assessed with and without the application of ZnO-NPs under different concentrations of Cd stress. Our findings indicate that increasing Cd levels significantly decreased sugar content at both 50 and 100 µM concentrations in both varieties. The reduction in soluble sugar was more pronounced at 100 µM Cd concentration in the NM-2011 variety compared to control plants. Additionally, a significant increase in sugar content was observed with the addition of ZnO-NPs (80 mg/L) under 100 µM Cd stress in the NM-2021 variety (Fig. 3).

### Antioxidants and ROS

When exposed to Cd stress, mung bean varieties showed a notable increase in the accumulation of total phenolic flavonoids, glycine betaine, and proline contents at a Cd concentration of 50 µM in both varieties. These contents further increased at a 100 µM Cd concentration compared to the control. In this study, we found that applying 80 mg/L ZnO-NPs was more effective in enhancing the phenolic, total flavonoids, glycine betaine, and proline contents in the NM-2021 variety (Fig. 3).

H_2_O_2_ and MDA levels serve as crucial indicators of oxidative stress in plants facing adverse environmental conditions. Our research indicates that, in comparison to the control group, exposure to Cd stress (50 and 100 µM) significantly increased the H_2_O_2_ and MDA concentrations in both types of mung beans. However, the application of ZnO-NP notably lowered the H_2_O_2_ and MDA levels in plants treated with Cd, thereby preserving the cellular membrane’s integrity. In the present study, applying ZnO-NPs (80 mg/L) at a Cd-stressed level of 100 µM markedly reduced H_2_O_2_ and MDA in the NM-2011 variety (Fig. 3).

### Nutrient uptake and root exudates

**Fig. 4:**
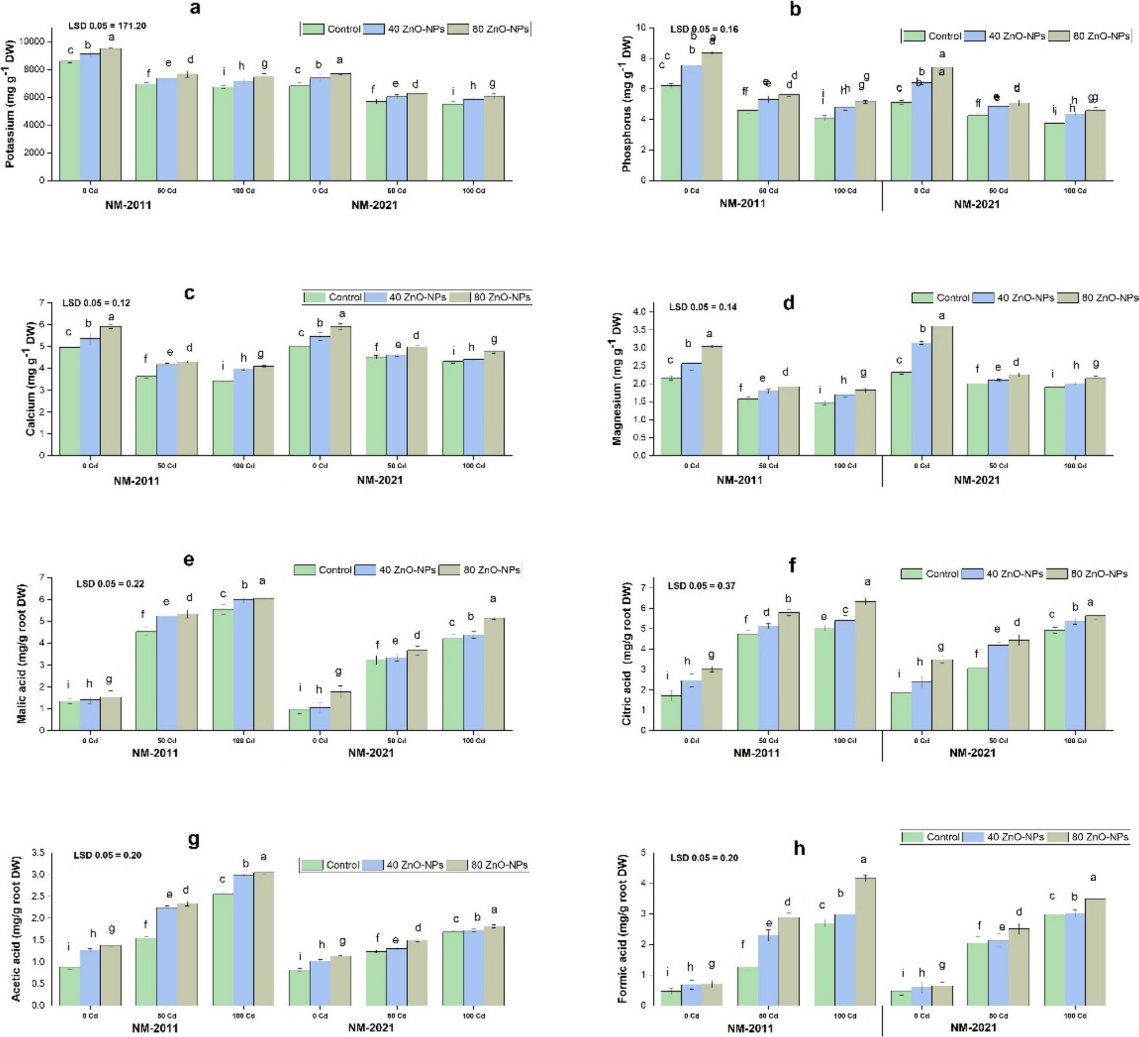
Impact of ZnO-NPs on nutrient uptake (**a**) potassium (**b**) phosphorus (**c**) calcium (**d**) magnesium and organic acids, including (**e**) malic acid (**f**) citric acid (**g**) acetic acid (**h**) formic acid of two mung bean genotypes NM-2011 and NM-2021 under cadmium stress

The research results indicated that the presence of Cd led to a reduction in essential nutrients in the mung bean. In the NM-2011 and NM-2021 varieties, the levels of K, P, Ca, and Mg decreased when exposed to Cd stress at concentrations of 50 and 100 µM. A more pronounced reduction was observed in the NM-2011 variety under 100 µM Cd stress compared to the control. Nevertheless, it was observed that applying 80 mg/L ZnO-NPs at a Cd stress level of 100 µM significantly enhanced nutrient absorption in the NM-2011 variety (Fig. 4).

The research indicated that as the concentration of Cd increased, the quantity of root exudates also increased. Various root exudates were assessed under different Cd stress conditions. When comparing plants grown without Cd stress to those subjected to 50 and 100 µM Cd treatments, there was a notable increase in the levels of malic acid, formic acid, acetic acid, and citric acid in the NM-2011 and NM-2021 varieties. However, it was observed that both applications of ZnO-NPs were more effective in alleviating Cd toxicity in both varieties studied. Additionally, our findings showed that ZnO-NPs (80 mg/L) further enhanced the root exudates in the NM-2011 variety under 100 µM Cd stress (Fig. 4).

### Water relations

**Fig. 5:**
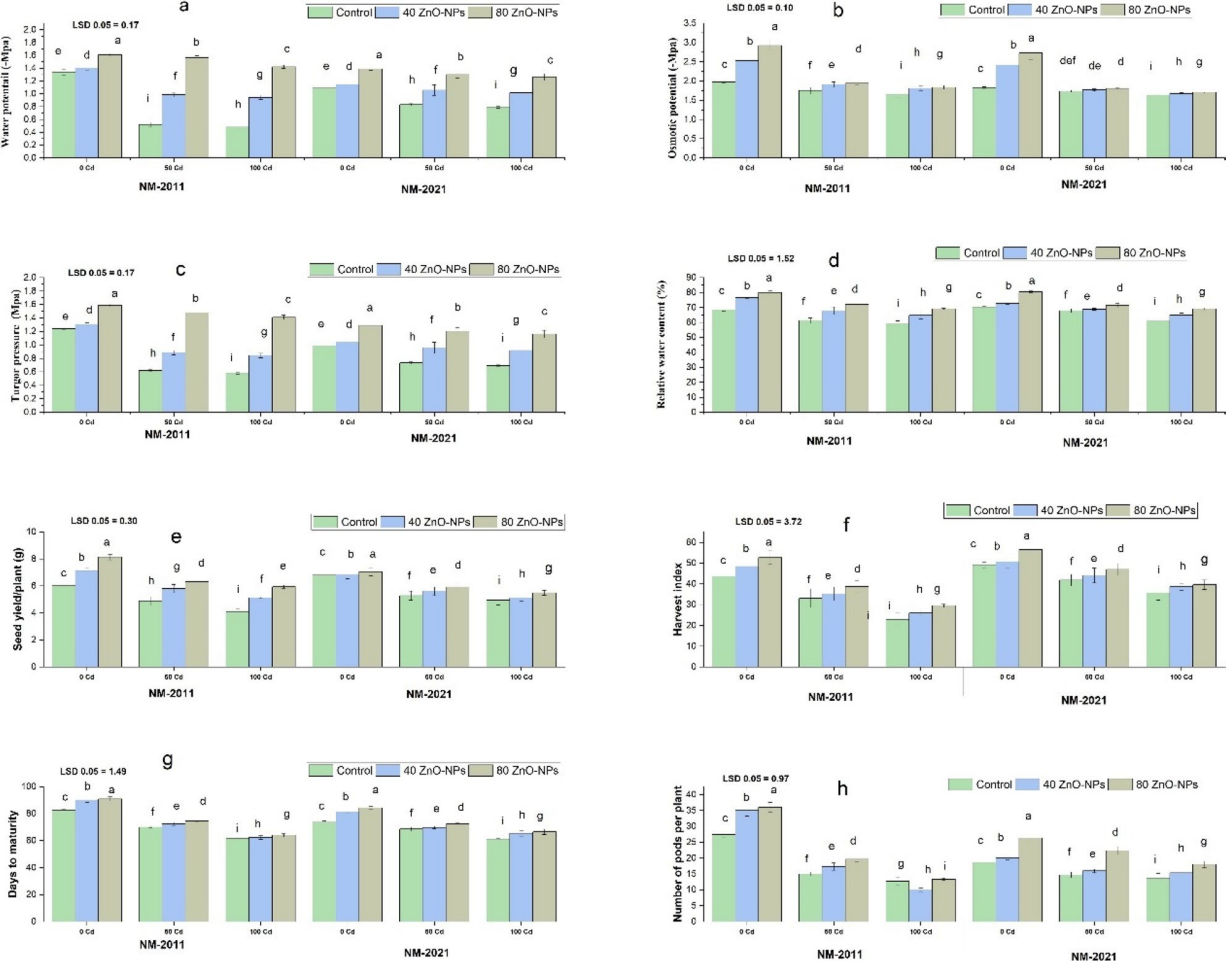
Evaluating various concentrations of ZnO-NPs on plant-water relations (**a**) Water potential (**b**) Osmotic potential (**c**) Turgor pressure (**d**) Relative water content and yield parameters (**e**) Seed yield per plant (**f**) Harvest index (**g**) Days to maturity (**h**) number of pods per plant of two mung bean varieties viz. NM-2011 and NM-2021 under the cadmium stress

A crucial element contributing to reduced growth under Cd stress is the presence of Cd in the soil, which disrupts the water balance and renders it inaccessible to plants. In the NM-2011 and NM-2021 varieties, water potential, pressure potential, osmotic potential, and relative water content diminished under Cd stress at 50 and 100 µM levels. When exposed to Cd 100 µM stress, the application of ZnO-NPs (80 mg/L) in the NM-2011 variety enhanced water relation attributes more significantly than in the NM-2011 variety (Fig. 5).

### Yield components

Elevated Cd levels significantly affected yield characteristics compared to plants grown without Cd stress. When compared to plants cultivated with 0 µM Cd, there was a notable reduction in seed yield per plant, harvest index, number of pods per plant, and days to maturity in the NM-2011 and NM-2021 varieties at 50 and 100 µM Cd concentrations. The application of ZnO-NPs at 40 and 80 mg/L levels enhanced seed yield per plant, harvest index, number of pods per plant, and days to maturity under 50 and 100 µM Cd stress in the NM-2011 and NM-2021 varieties. At 100 µM Cd, applying ZnO-NPs at an 80 mg/L concentration further improved the yield components in the NM-2011 variety compared to the NM-2021 variety (Fig. 5).

### PCA Analysis

A biplot was further constructed to provide an overview of relationships using the principal component analysis (PCA). Dim 1 and Dim 2 are the first two components that keep a lot of total variance (Dim 1 = 056.5%, Dim 2 = 19.6%). The biplot well segregates the variables into two principal functional groups along the horizontal axis (Dim1). The stress responses are significantly associated with the variables on the right side of the plot. This cluster also includes CAT, APX, SOD, and POD, which are major antioxidant enzymes, and the Osmoprotectant like Proline (P) and Glycine Betaine [GB]. Moreover, this region contained indicators of oxidative damage, H_2_O_2,_ and MDA. This indicates that the positive direction of Dim 1 is a reflection of defense and stress-related changes in plant metabolism.

**Fig. 6:**
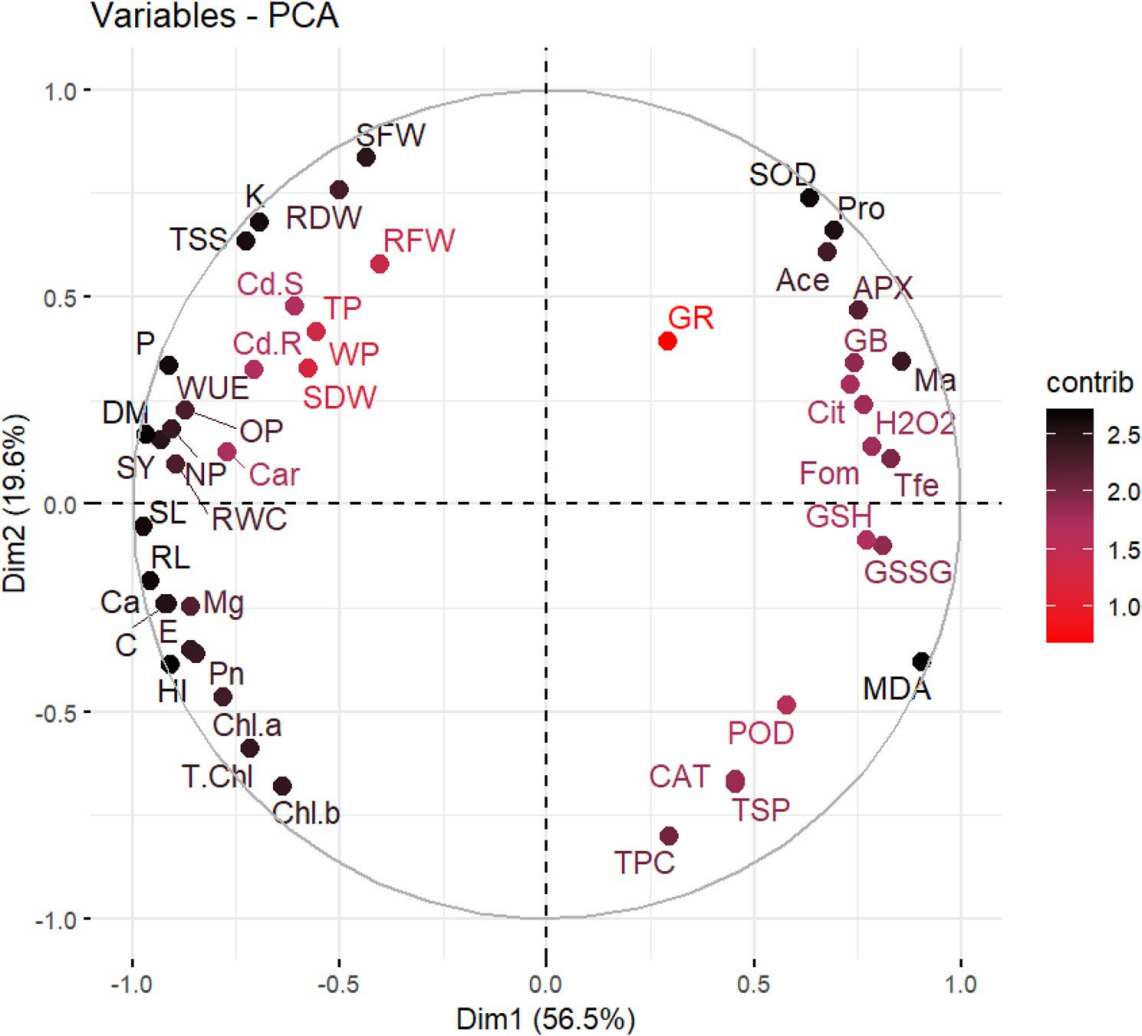
Principal Component Analysis (PCA) biplot for evaluation of zinc oxide nanoparticles on cadmium-stressed mung bean cultivars, focusing on various agronomic, physiological, and biochemical traits. Different abbreviations used in this figure are as follow: RL, root length; SL, shoot length; SFW, shoot fresh weight; SDW, shoot dry weight; RFW, root fresh weight; RDW, root dry weight; Chl a, chlorophyll a; Chl b, chlorophyll b; T. Chl, total chlorophyll; Car, carotenoids; SOD, superoxide dismutase; POD, peroxidase activity; CAT, catalase; APX, ascorbate peroxidase; MDA, malondialdehyde content; GB, glycine betaine, Fle, flavonoids; Phe, phenolics; H_2_O_2_, hydrogen peroxide; Pro, proline, TSP, total soluble protein; TSS, total soluble sugar; Ca, calcium; Mg, magnesium; K, potassium; P, phosphorus; Ma, malic acid; Cit, citric acid; Ace, acetic acid, For, formic acid; Cd R, cadmium contents in roots; Cd S, cadmium contents in shoots

In contrast, the variables on the left side of the plot are related to growth and yield parameters and nutrient content in the plant. This group consists of traits like DM (Dry Matter), SY (Seed Yield), RL (Root Length), and macro minerals; K, P, Ca, and Mg. The strong negative correlation between these two sets of variables along Dim1 is a fundamental result, because traits related to growth and yield decrease as the values related to stress increase, which is a typical physiological plant response. Dim2 (the vertical axis) is great to separate stress responses from growth metrics. As an example, those variables associated with T.Chl activity (T.Chl, Chl. b) negative correlation between stress markers (MDA, TPC). This is consistent with Dim2 being a second dimension of response, likely because in some way related to specific metabolic pathways or nutrient partitioning under stress (Fig. 6).

### Relationship among tested variables

**Fig. 7:**
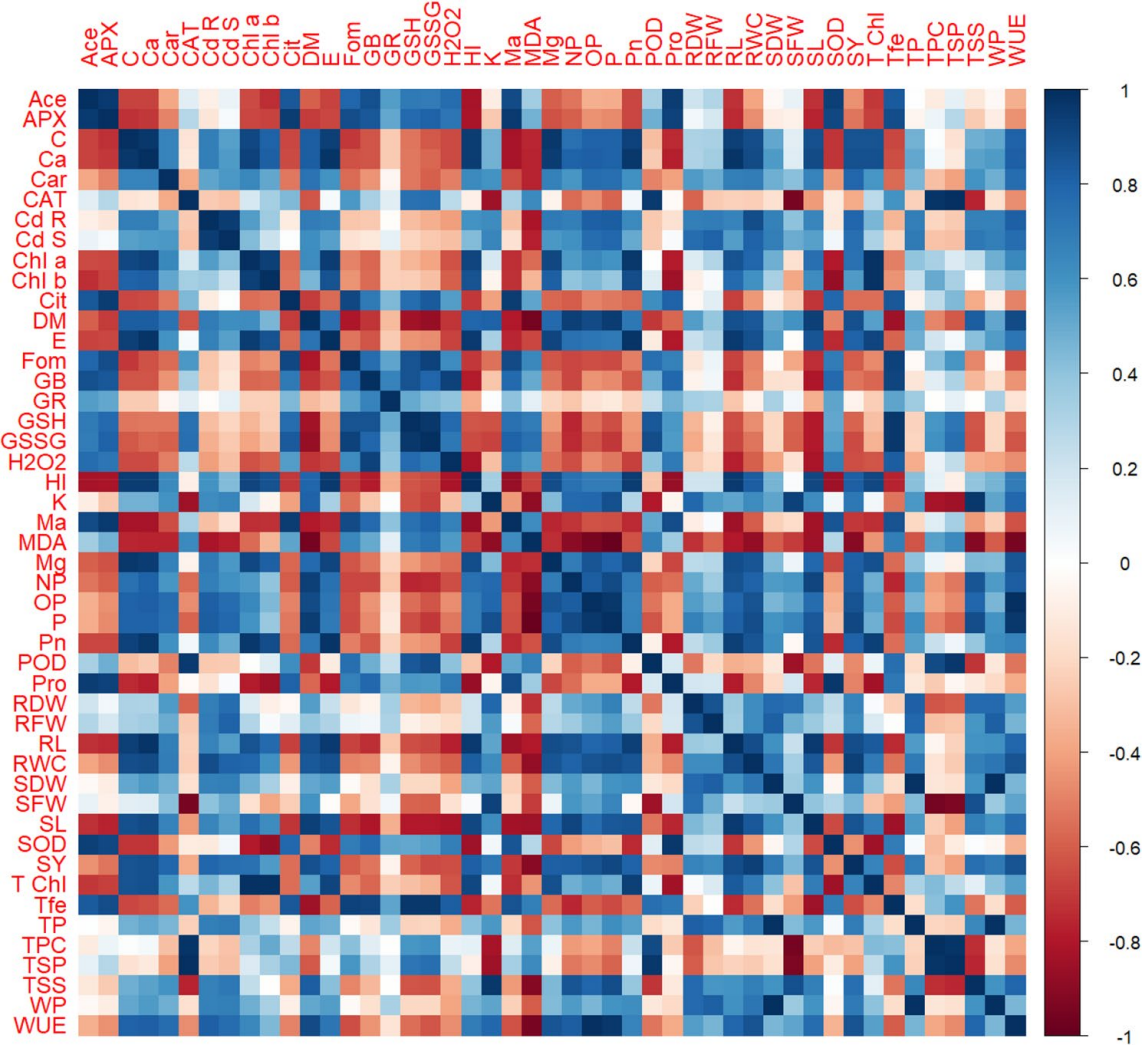
Correlation analysis of various agronomic, physiological, and biochemical traits for evaluation of zinc oxide nanoparticles on cadmium-stressed mung bean cultivars. In the correlation matrix, all pairs of variables have their Pearson correlation coefficients visualized. The color intensity and direction show the correlation of strength: Dark blue with + 1 means strong positive correlation, Dark red with − 1 means strong negative correlation, and white with 0 means no or weak correlation. The diagonal indicates a perfect correlation of 1 for each variable with itself. Different abbreviations used in this figure are as follow: RL, root length; SL, shoot length; SFW, shoot fresh weight; SDW, shoot dry weight; RFW, root fresh weight; RDW, root dry weight; Chl *a*, chlorophyll *a*; Chl *b*, chlorophyll *b*; T. Chl, total chlorophyll; Car, carotenoids; SOD, superoxide dismutase; POD, peroxidase activity; CAT, catalase; APX, ascorbate peroxidase; MDA, malondialdehyde content; GB, glycine betaine, Fle, flavonoids; Phe, phenolics; H_2_O_2_, hydrogen peroxide; Pro, proline, TSP, total soluble protein; TSS, total soluble sugar; Ca, calcium; Mg, magnesium; K, potassium; P, phosphorus; Ma, malic acid; Cit, citric acid; Ace, acetic acid, For, formic acid; Cd R, cadmium contents in roots; Cd S, cadmium contents in shoots

The correlation matrix shown in Fig. 7 allows identification of strong Positively Correlated variable groups and Negatively Correlated pairs within the observed data. The most common observation is the good positive correlation with plant growth and yield parameters. By contrast, dry matter (DM), seed yield (SY), and several nutrients such as K, P, Ca, and Mg all stand out here with very high positive correlation, suggesting that large root systems can well access and intercept the available nutrients in soil to enhance plant growth and seed yield. Also, water potential (WP), osmotic potential (OP), and turgor pressure (TP) are all related to water relation variables, displaying a great physiologic interdependence of the plant in its water regime.

Conversely, an entirely different set of variables across the spectrum exhibits a strong positive correlation, led for the most part by stress response markers. In addition to antioxidant enzymes (SOD, POD, CAT), osmolytes including proline (Pro) and glycine betaine (GB), as well as the essential indicators of oxidative damage hydrogen peroxide (H_2_O_2_​) and malondialdehyde (MDA), accounted for this cluster. This group of positive correlations is largely intuitive because the hormonal crosstalk and the generation of ROS occurring in a stressed plant is extensive to elicit defensive responses, leading nevertheless to damage of cellular entities. The strongest negative coupling from the matrix is between the growth/yield group and the stress response group. It is this antagonistic relationship that we were talking about, which can easily be visualized in the heatmap, where dark red blocks clearly show a strong negative association. High levels of MDA and H_2_O_2_​ adversely affect the seed yield (SY) and dry matter (DM). The strong negative correlation underscores the well-known tradeoff between growth and defense, since plants facing stress are forced to allocate their limited resources toward survival and defenses, which necessarily reduces growth and productivity. This pattern is dominant across plant physiology and therefore does seem to agree well with what we can visualize in the correlation matrix.

Cd levels were highest in the root organs relative to the shoots. Cd stress (50µM) increased the Cd ion in the root and shoot of NM-2011 and NM-2021 mung bean varieties, respectively. Further increase was also observed in Cd ion in root and shoot at 100 μm Cd concentration in NM-2011 and NM-2021 varieties, in contrast to the control. ZnO-NPs treatment substantially decreased the levels of Cd ions in the root and shoot. ZnO-NPs applied at 80 mg/L were more beneficial in decreasing Cd level in root/shoot at 100 µM Cd stress in the NM-2011 variety (Table 1).

**Table 1:** The effect of different concentrations of ZnO-NPs on Cd contents in roots and shoots of mung bean (*Vigna radiata* L.) under Cd stress

		NM-2011		NM-2021	
Treatments	Cd levels	Cd content in the root (mg/kg)	Cd content in the shoot (mg/kg)	Cd content in the root (mg/kg)	Cd content in the shoot (mg/kg)
Control	0 Cd	30.21 ± 0.83c	12.55 ± 1.21c	24.45 ± 1.18c	8.78 ± 1.18c
	50 Cd	46.40 ± 1.87 g	19.21 ± 0.84f	42.41 ± 1.14f	17.74 ± 0.84 g
	100 Cd	73.45 ± 0.91i	37.56 ± 0.93i	69.88 ± 1.06i	26.55 ± 0.45i
40 mg/L ZnO-NPs	0 Cd	23.45 ± 6.90b	8.45 ± 2.49b	20.14 ± 1.70b	7.47 ± 1.64b
	50 Cd	37.74 ± 1.93e	18.74 ± 2.85e	31.63 ± 2.29d	15.96 ± 1.69f
	100 Cd	56.40 ± 1.01 h	26.40 ± 1.01 h	53.39 ± 2.18 g	18.06 ± 1.20 h
80 mg/L ZnO-NPs	0 Cd	17.05 ± 3.94a	6.02 ± 1.80a	15.48 ± 3.27a	4.68 ± 1.18a
	50 Cd	34.07 ± 1.25d	17.40 ± 2.62d	24.36 ± 2.73c	11.69 ± 0.72d
	100 Cd	39.00 ± 3.50f	20.67 ± 1.25 g	33.94 ± 1.51e	14.94 ± 1.51e

In the current study, a number of antioxidant enzymes were also examined in mung bean cultivars. According to the current results, SOD, POD, CAT, and APX were raised to the Cd level of 50 and 100 µM Cd in NM-2011 and NM-2021, in contrast to the control. Antioxidant enzymes in this study responded differently as ZnO NP concentrations increased. The used NPs have the capacity to enhance the system’s ROS production. Under Cd (100 µM) stress, the level of ZnO-NPs (80 mg/L) increases more antioxidant enzymes in the NM-2021 variety as compared to the NM-2011 variety (Table 2).

**Table 2:** Enzyme activities of mung bean (*Vigna radiata* L.) through different concentrations of ZnO-NPs under Cd stress

Parameters	Varieties	Cd levels (µM)	Control	40 mg/L ZnO-NPs	80 mg/L ZnO-NPs
Leaf SOD activity (Units mg^− 1^ protein)	NM-2011	0 Cd	172.73 ± 1.49a	175 ± 2.32b	177 ± 2.83c
		50 Cd	211 ± 2.87d	213 ± 2.59e	230 ± 2.87f
		100 Cd	241 ± 2.85 g	243 ± 2.56 h	260 ± 2.81i
	NM-2021	0 Cd	236 ± 2.29a	239 ± 3.76b	249 ± 2.32c
		50 Cd	250 ± 3.44d	259 ± 2.87e	268 ± 2.21f
		100 Cd	280 ± 3.40 g	289 ± 2.83 h	298 ± 2.01i
Leaf POD activity (Units mg^− 1^ protein)	NM-2011	0 Cd	9855 ± 403a	10,488 ± 284b	10,955 ± 319c
		50 Cd	11,355 ± 364d	11,988 ± 306e	12,490 ± 326f
		100 Cd	13,355 ± 364 g	13,988 ± 306 g	14,490 ± 326 h
	NM-2021	0 Cd	12,384 ± 345a	12,957 ± 415a	13,017 ± 392b
		50 Cd	13,652 ± 303bc	14,054 ± 316d	14,959 ± 334de
		100 Cd	15,652 ± 303e	15,987 ± 309f	16,959 ± 340 g
Leaf CAT activity (Units mg^− 1^ protein)	NM-2011	0 Cd	320 ± 22a	375 ± 27b	398 ± 14c
		50 Cd	436 ± 23d	460 ± 18e	486 ± 19f
		100 Cd	516 ± 23 g	540 ± 18 h	566 ± 19i
	NM-2021	0 Cd	560 ± 22a	567 ± 23ab	571 ± 23bc
		50 Cd	591 ± 21d	602 ± 21e	631 ± 24f
		100 Cd	671 ± 21 g	682 ± 21 h	711 ± 24i
Leaf APX activity (Units mg^− 1^ protein)	NM-2011	0 Cd	560 ± 22a	606 ± 23b	703 ± 20c
		50 Cd	770 ± 17d	820 ± 25e	868 ± 21f
		100 Cd	895 ± 17 g	930 ± 26 h	978 ± 21i
	NM-2021	0 Cd	570 ± 28a	603 ± 20b	629 ± 24bc
		50 Cd	662 ± 14d	701 ± 24e	732 ± 18f
		100 Cd	772 ± 16 g	811 ± 24 h	842 ± 18i
Leaf GR Content (Units mg^− 1^ protein)	NM-2011	0 Cd	12.89 ± 1.04a	13.61 ± 1.06ab	14.17 ± 1.45c
		50 Cd	18.98 ± 1.45d	22.96 ± 1.49e	24.46 ± 1.75ef
		100 Cd	35.86 ± 1.62f	36.4 ± 1.48 g	39.4 ± 0.95 h
	NM-2021	0 Cd	16.36 ± 1.21a	16.41 ± 1.16ab	18.08 ± 0.60bc
		50 Cd	20.12 ± 1.23d	24.42 ± 2.10e	26.92 ± 1.34f
		100 Cd	33.53 ± 0.86 g	34.64 ± 0.55 h	38.001 ± 2.02i
Leaf GSSG Content (mg g^− 1^ FW)	NM-2011	0 Cd	16.40 ± 1.12a	17.73 ± 1.24b	18.99 ± 0.88c
		50 Cd	24.16 ± 1.53d	24.83 ± 1.44e	27.98 ± 1.38f
		100 Cd	34.65 ± 1.52 g	36.98 ± 1.42 h	38.65 ± 0.81i
	NM-2021	0 Cd	21.41 ± 1.06a	22.75 ± 0.82ab	24.30 ± 0.32b
		50 Cd	27.45 ± 1.73c	29.09 ± 0.72d	30.09 ± 0.92ef
		100 Cd	35.59 ± 0.98f	37.26 ± 1.08 g	39.00 ± 1.50 h
Leaf GSH content (mg g^− 1^ FW)	NM-2011	0 Cd	0.92 ± 0.07a	1.008 ± 0.077b	1.12 ± 0.09c
		50 Cd	1.21 ± 0.11d	1.34 ± 0.02e	1.37 ± 0.08f
		100 Cd	1.75 ± 0.08 g	1.80 ± 0.10 h	1.92 ± 0.07i
	NM-2021	0 Cd	1.31 ± 0.065a	1.33 ± 0.05ab	1.36 ± 0.06bc
		50 Cd	1.44 ± 0.055d	1.46 ± 0.07de	1.50 ± 0.05e
		100 Cd	1.64 ± 0.048f	1.66 ± 0.10 g	1.75 ± 0.11 h

Cd stress in plants causes drastic changes in enzyme activity due to oxidative stress. In comparison to plants grown with 0 µM Cd, a significant rise in glutathione contents, such as GR, GSSG, and GSH content, was observed in NM-2011 and NM-2021 varieties at 50 and 100 µM Cd levels. ZnO-NPs applied at 40, 80 mg/L levels also increased GR, GSSG, and GSH content at 50 and 100 µM Cd stress in NM-2011 and NM-2021 varieties, while at 100 µM Cd, the level of ZnO-NPs applied at 80 mg/L concentration further increased the GR, GSSG, and GSH content in the NM-2021 variety as compared to the NM-2011 variety (Table 2).

## Discussion

Co-existence and interaction of many elemental nanoparticles is an area of growing interest in many environmental systems [47]. As there could be an interplay between heavy metals and these nanoparticles for absorbing and accumulating in different plant tissues [48]. At the same time, these studies become more critical as heavily metal-polluted food crops are the major pathway of their entrance into human bodies [49]. Nanotechnology is being exploited in agriculture to deal with various abiotic and biotic stressors experienced by different crop species under rapidly changing climatic conditions [50, 51]. Previous research has shown that application of Zn nanoparticles efficiently modifies the accumulation level of different heavy metals, including Cd, in many plant species, thus reducing their toxicity [52].

Cadmium is unnecessary for plants because its toxic effects result in general developmental abnormalities [53]. The current study showed that roots become necrotic, rotting, and mucilaginous following prolonged Cd exposure, restricting plant shoot and root elongation [14]. Cadmium exposure lowers the number of meristematic cells, resulting in decreased dry biomass and root length while increasing root diameter [54]. Cd exposure reduces the elongation of roots in crops like rice, tomato, and wheat [14]. Zn is released by the NPs of ZnO, an essential micronutrient that regulates plant growth [55]. The involvement of Zn in auxin production is widely known, and this hormone is involved in cell expansion and cell division, which leads to plant growth [3]. We also noted in this study that ZnO-NPs significantly reversed the toxic effects of Cd in both mung bean cultivars. Cell division and expansion possibly increased all studied growth parameters.

Cd exposure had a substantial effect on the production of photosynthetic pigments in mung bean cultivars in the current study. The harmful impact of Cd stress on photosynthetic pigments of plants is the consequence of enhanced production of chlorophyllase, which is thought to alter the carotenoids and chlorophyll synthesizing process and induce chlorophyll breakdown [56, 57]. The ZnO-NPs accumulation in the present study was observed to be a positive stimulator of chlorophyll and carotenoid synthesis, resulting in better photosynthetic plant efficiency as manifested by enhanced biomass [58]. Furthermore, Zn is required for the action of carbonic anhydrase, which mediates CO_2_ hydration to bicarbonate for transport to chloroplasts [59], and we observed that ZNO-NPs application increased CO_2_ assimilation, thus increasing net photosynthesis and thereby increasing the plant biomass.

According to recent study Cd impacts plant gas exchange characteristics via stomatal closure, CO_2_ intake decrease, and water relations [60]. Furthermore, ROS formation in Cd stress might damage the chloroplast ultrastructure and thylakoid membrane, resulting in a decrease gas exchange attribute [61]. The photosynthetic machinery, notably photosystems I and II and the light-harvesting complex is damaged by cadmium [62]. Iron (Fe) helps in the improvement of chlorophyll concentration and the production of many other pigments directly engaged in photosynthesis light harvesting [54]. Blockage of iron reductase by cadmium results in iron deficit, which negatively affects the photosynthetic process [63]. The use of ZnO-NPs improved gas exchange properties [64]. NPs boost photosynthetic rate by increasing electron transport chain and water photolysis [65, 66].

Cadmium ions disrupt protein functions by modifying their structure and replacing metal cofactors for example Ca and Zn ions [67]. Zinc has been shown to boost the efficiency of ionic inter-conversions that promotes nitrogen intake and results in increased content of protein [68, 69]. Same findings have already been documented by Tripathi et al. [70]. Application of ZnO-NP on the other hand, enhanced protein production [71].

Exposure to Cd activates some specific biosynthetic pathways and so increases the content of phenolics and flavonoids. Essential to this process is regulation of key enzymes in flavonoid biosynthesis like chalcone synthase (CHS) and flavonoid 3′-hydroxylase (F3H). These enzymes promote to the transformation from phenolic precursors, to all sort of flavonoid structures which protect against cadmium toxicity [72]. More importantly treatment of zinc oxide nanoparticles (ZnO-NPs) increased the total flavonoid content through a possible regulation on the expression of flavonoid biosynthesis genes; snapdragon plants can overcome cadmium-induced stress [73, 74].

According to current research, Cd exposure increases proline contents and GB concentrations in mung bean plants [75]. Plant adaptability, tolerance and protection against Cd stress are aided by proline and GB [76]. Plants accumulate proline as a mechanism to combat Cd stress by changing osmotic potential, stabilizing membrane structures, and reducing stress [77]. Furthermore, proline’s protective activity involves the generation of nontoxic Cd-proline group, functioning as a C and N source, and triggering the antioxidant system [78]. According to Torabian et al. [79]. GB aids in the maintenance of the plant’s glyoxalase and antioxidant systems, hence increasing stress resistance. ZnO-NP appears to stimulate the GB and proline biosynthesis pathways in plants, which could be beneficial for reducing Cd stress [80, 81].

Free radicals formed by ROS (H_2_O_2_) following Cd exposure are a significant factor that influences the toxicity of Cd on several plants [82]. In general, these reactive molecules are generated when Cd interacts with the -SH functional groups and antioxidants [78]. It causes lipid peroxidation, which results in MDA overproduction. These findings imply that Cd caused lipid peroxidation degrades membrane structures that play an important role in plant metabolism maintenance [83]. The supply of ZnO-NPs against Cd exposed plants lowered MDA and H_2_O_2_ by regulating cellular metabolism, which is a significant finding in the current work [84].

GSH is the more prevalent sulfydryl-containing non-protein molecule that is essential for maintaining homeostasis, cellular signaling, redox regulation and the defence system of plants [85]. It functions as a redox pair along with its reduced (GR) and oxidized forms (GSSG), playing a significant part in a variety of signaling systems in plants [86, 87]. Nair and Chung [88] suggested that Cd binds to antioxidant enzymes thiol groups, which has a direct impact on biochemical reactions and inhibits plant development in general [89]. In present study, the Cd-stressed mung bean plants’ SOD, APX, POD, CAT, GSH, GR, and GSSG activities were increased by ZnO-NP [90]. The plants were able to scavenge the ROS produced in reaction to stressful stimuli thanks to the antioxidants’ improved activity [91].

Cd ions with in soil can interfere with root absorption as well as the subsequent transportation of nutrients in mung bean [92]. Cd exposure considerably reduces the N, Ca, Mg, and P levels of mung bean [53]. Under extreme Cd stress, reduce in root length and surface area are associated with Cd stress that imply lower resource storage potential [93]. The transfer of micro and macronutrients of poplar plant (*Populus acquemontiana* L.) is hampered by competition for important nutrients with Cd [94]. ZnO-NP may affect the nutrients homeostasis and subsequently metabolism, particularly nitrogen assimilation, through mechanisms involving alteration in the membrane transporters expression, differentiation of the vascular system, hormonal balances, and source/sink relationships [95].

Cd-induced high root organic acid contents seem to mainly serve detoxification in mung bean roots. This could be due to low-molecular-weight organic acids of plant root exudates that can chelate Cd ions, and result in the formation of stable complexes between Cd ions and carboxyl or amino groups, which restricted the mobility of Cd within plants bodies and lower the transport capability from roots to shoots. Hence, while exudation of organic acids could lead to an apparent increase in Cd solubilization in soil early during plant growth, the most important effect in our study was likely internal immobilization and sequestration of Cd within cell vacuoles [96]. ZnO-NP also considerably increased the amount of different organic acids because of production of metal-ion complexes inside the root of the mung bean; the organic acids that have been released may provide the plant with better protection by reducing metal transport to the shoots [97–99].

Cd contamination in roots causes reduced water absorption as well as inhibition of short-distance water transport [100]. The reduction of aquaporin functions and variations in protein expression are most likely to be responsible for the impaired water transport through the membrane [101]. However, there was a notable improvement in the water potential and RWC of the leaves when Cd-stressed soybean plants were supplied with various levels of ZnO-NP. This might have happened because ZnO-NP increased the absorption of mineral and water elements [102].

In mung bean shoots and roots, the concentration of Cd increased with elevated Cd levels, which correlated with heightened rhizospheric acidity at these higher Cd concentrations. The enhanced uptake of apoplastic Cd results in increased Cd content in the plant, and as Cd treatment levels rise in mung beans, there is a corresponding increase in Cd accumulation within root cells [103]. Areas of the roots directly exposed to Cd exhibited signs of increased endodermal growth [104]. Similarly, the application of ZnO affects both the uptake and transport of Cd through different mechanisms: in the rhizosphere, ZnO‑NPs adsorb Cd ions on their surface, reducing their Phyto availability, likely due to the formation of (Cd, Zn) complexes; additionally, the released Zn may compete with Cd for uptake sites, limiting its absorption. This dual mechanism can explain the reduction in Cd accumulation and the improved stress tolerance observed in mung beans [105].

Mung bean yield characteristics were severely affected by cadmium toxicity. This yield reduction was attributed to increased level of oxidative stress, formation of MDA and H_2_O_2_, Cd absorption, and sharp declines in photosynthetic pigments and RWC levels. When ZnO-NPs were added, the yield attributes were significantly improved both in normal and in Cd-stressed conditions. The improved yield characteristics resulted from ZnO-NPs’ protective effects on proteins and cell membranes, enhanced chlorophyll synthesis, and increased antioxidant activities [106].

## Conclusion

This study was conducted to investigate the mitigation in Cd-stressed mung bean (*Vigna radiata* L.) using zinc oxide nanoparticles (ZnO-NPs). Two varieties of mung bean, namely NM-2011 and NM-2021, were treated with 40 mg/L and 80 mg/L ZnO-NPs through foliar spray after exposing them to varying levels (50µM and 100µM) of Cd. The findings deduced that ZnO-NPs substantially ameliorated the inhibitory effects of Cd stress on plant growth, photosynthetic parameters, antioxidant enzymes, and yield attributes in both mung bean varieties. ZnO-NPs at the 80 mg/L concentration was determined to be the most effective treatment for reducing Cd toxicity. With ZnO-NPs, water uptake was increased (water potential 84% increase, turgor pressure 72% increase, osmotic potential 11%, RWC 16%), antioxidant enzyme activities (SOD 10%, POD 8%, CAT 9%, APX 37%, GR 13%, GSSG 9%, GSH 6%) were enhanced, oxidative stress was alleviated, whereas photosynthetic performance under Cd stress followed an opposite pattern (total chlorophyll 39% increase, chlorophyll a 38% increase, chlorophyll b 41% increase, carotenoids 40% increase, net photosynthetic rate 92% increase, stomatal conductance 30% increase, transpiration rate 73% increase, WUE 11% increase). Total soluble proteins and sugars exhibited an increase of 8% and 25%, respectively. Total phenolics and flavonoids, GB and proline showed an increasing trend of 9%, 12%, 14% and 5%, respectively. Lipid peroxidation was decreased as MDA and H_2_O_2_ showed 15% and 18% decrease as compared to control. Vital nutrients exhibited increasing trend as K, P, Ca, and Mg showed 11%, 26%, 19% and 24% increase as compared to control. Organic acids such as malic acid (7% increase), formic acid (11% increase), acetic acid (17% increase) and citric acid (7% increase) showed enhanced levels as compared to control. Yield parameters also exhibited increasing trend as Seed yield per plant (44%), harvest index (28%), number of pods per plant (31%), and days to maturity (9%) as compared to control. This work reveals that nanotechnology-based approaches can be employed to promote heavy metal stress tolerance in plants for smart and sustainable agriculture. Although our results demonstrate improved physiological parameters with ZnO-NP treatment, future studies should explore nanoparticle bioaccumulation, long-term soil effects, and gene expression pathways involved in Cd detoxification.

## Supplementary Information

Below is the link to the electronic supplementary material.

Supplementary Material 1.

## Data Availability

All the data and materials used in this study has been mentioned in the manuscript.

## Abbreviations

Cd: Cadmium
ZnO NPs: Zinc Oxide Nanoparticles
NM-2011: NIAB Mung 2011
NM-2021: NIAB Mung 2021
SOD: Super Oxide Dismutase
POD: Peroxidase
CAT: Catalase
APX: Ascorbate Peroxidase
TPC: Total Phenolic Contents
TFC: Total Flavonoid Contents
GB: Glycine Betaine
H_2_O_2_: Hydrogen Peroxide
MDA: Malondialdehyde
GR: Reduced Glutathione
GSSG: Oxidized Glutathione
ROS: Reactive Oxygen Species
