# Supplementary material for: Modulation of antioxidant systems and photosynthetic machinery by foliar-applied ZnO nanoparticles in cadmium-stressed mung bean (Vigna radiata L.)
Source: BMC Plant Biol. 2026 Mar 11;26:700. doi: 10.1186/s12870-026-08452-7 (PMC13088788; doi:10.1186/s12870-026-08452-7)
Supplement: Supplementary file 1 — Supplementary Material 1. [file 12870_2026_8452_MOESM1_ESM.docx]

**Table 1. ANOVA for evaluating the impact of cadmium stress on two mung bean cultivars NM-2011 and NM-2021 via zinc oxide nanoparticle application and their interactive effect on growth and biomass.**

|  |  | **Shoot Length** | **Root Length** | **Shoot Fresh Feight** | **Shoot Dry Weight** | **Root Fresh Weight** | **Root Dry Weight** |
| --- | --- | --- | --- | --- | --- | --- | --- |
| **Source** | **df** | **MS** | **MS** | **MS** | **MS** | **MS** | **MS** |
| **Main Effects** |  |  |  |  |  |  |  |
| **Stress** | 2 | 417.92 *** | 160.68 *** | 9.55*** | 0.34* | 0.11* | 0.01** |
| **Treatments** | 2 | 47.98 *** | 30.74 *** | 0.36ns | 1.35*** | 0.29*** | 0.02*** |
| **Varieties** | 1 | 8.32ns | 30.67 *** | 70.06*** | 0.45** | 0.13** | 0.06*** |
| **Interaction** |  |  |  |  |  |  |  |
| **stress × Treatments** | 4 | 41.57 *** | 0.55ns | 5.38ns | 0.23* | 0.06* | 0.02*** |
| **stress × Varieties** | 2 | 68.82 *** | 21.92 *** | 0.91ns | 0.31* | 0.05* | 0.01*** |
| **Treatments × Varieties** | 2 | 32.06 *** | 0.68 | 0.14ns | 0.48*** | 0.08** | 0.04*** |
| **Stress × Treatments × Var** | 4 | 28.15 ** | 0.31ns | 0.04ns | 0.24* | 0.010* | 0.02*** |
| **Error** | 36 | 3.91 | 1.00ns | 1.00 | 0.09 | 0.013 | 0.01 |
| **Total** | 53 |  |  |  |  |  |  |

**Table 2. ANOVA for evaluating the impact of cadmium stress on two mung bean cultivars NM-2011 and NM-2021 via zinc oxide nanoparticle application and their interactive effect on photosynthesis and gas exchange parameters**

|  |  | **Chlorophyll a** | **Chlorophyll b** | **Total Chlorophyll** | **Carotenoids** | **Net Photosynthetic rate** | **Stomatal Conductance** | **Transpiration Rate** | **Water Use Efficiency** |
| --- | --- | --- | --- | --- | --- | --- | --- | --- | --- |
| **Source** | **Df** | **MS** | **MS** | **MS** | **MS** | **MS** | **MS** | **MS** | **MS** |
| **Main Effects** |  |  |  |  |  |  |  |  |  |
| **Stress** | 2 | 73154*** | 69982 *** | 286236*** | 532.11*** | 247*** | 103*** | 74*** | 2.42*** |
| **Treatments** | 2 | 29326*** | 12109 *** | 76477*** | 180*** | 115*** | 20*** | 32*** | 0.72*** |
| **Varieties** | 1 | 121923*** | 276306 *** | 765317*** | 1.45* | 248*** | 31*** | 66*** | 0.18* |
| **Interaction** |  |  |  |  |  |  |  |  |  |
| **stress × Treatments** | 4 | 64121*** | 734*** | 3132ns | 3.23*** | 0.46* | 22* | 1.40*** | 0.32*** |
| **stress × Varieties** | 2 | 53471** | 823 *** | 4277** | 3.01*** | 24*** | 41*** | 9.98*** | 0.23** |
| **Treatments × Varieties** | 2 | 53371** | 6108*** | 14800ns | 4.48*** | 5.12*** | 44*** | 0.92*** | 0.31*** |
| **Stress × Treatments × Var** | 4 | 68992 *** | 974*** | 3729** | 2.24** | 1.37*** | 32** | 0.21** | 0.43*** |
| **Error** | 36 | 2533 | 11.90 | 2693 | 1.09 | 0.15 | 0.47 | 0.06 | 0.02 |
| **Total** | 53 |  |  |  |  |  |  |  |  |

**Table 3. ANOVA for evaluating the impact of cadmium stress on two mung bean cultivars NM-2011 and NM-2021 via zinc oxide nanoparticle application and their interactive effect on biochemical indicators**

|  |  | **Total Soluble Protein** | **Total Soluble Sugar** | **Total Phenolics Content** | **Total Flavonoids Content** | **Proline Content** | **Glycine Betaine (GB)** | **Hydrogen Peroxide (H_2_O_2_)** | **Malondialdehyde (MDA)** |
| --- | --- | --- | --- | --- | --- | --- | --- | --- | --- |
| **Source** | **df** | **MS** | **MS** | **MS** | **MS** | **MS** | **MS** | **MS** | **MS** |
| **Main Effects** |  |  |  |  |  |  |  |  |  |
| **Stress** | 2 | 42354*** | 3197*** | 9.16*** | 6026*** | 7.96*** | 61*** | 56*** | 66734*** |
| **Treatments** | 2 | 3988*** | 1039*** | 1.07*** | 196*** | 0.19*** | 9.26** | 1.56** | 19012*** |
| **Varieties** | 1 | 145912*** | 366 *** | 8.69*** | 186** | 12.68*** | 11.33*** | 9.76*** | 19534*** |
| **Interaction** |  |  |  |  |  |  |  |  |  |
| **stress × Treatments** | 4 | 30.16ns | 641*** | 16.07* | 323** | 0.03*** | 22.01*** | 0.59* | 2058*** |
| **stress × Varieties** | 2 | 58.07ns | 788 *** | 18.05* | 455*** | 2.04*** | 23.06*** | 21.77*** | 1693** |
| **Treatments × Varieties** | 2 | 66.07ns | 289* | 63.13*** | 448*** | 0.06*** | 21.43** | 0.47* | 1506* |
| **Stress × Treatments × Var** | 4 | 89.46ns | 366** | 55.42** | 324** | 0.03*** | 28.91** | 0.58* | 1897*** |
| **Error** | 36 | 202 | 37.15 | 44.7 | 14.18 | 0.04 | 0.41 | 0.22 | 252 |
| **Total** | 53 |  |  |  |  |  |  |  |  |

**Table 4. ANOVA for evaluating the impact of cadmium stress on two mung bean cultivars NM-2011 and NM-2021 via zinc oxide nanoparticle application and their interactive effect on nutrient uptake and root exudates**

|  |  | **Potassium** | **Phosphorus** | **Calcium** | **Magnesium** | **Malic Acid** | **Citric Acid** | **Acetic Acid** | **Formic Acid** |
| --- | --- | --- | --- | --- | --- | --- | --- | --- | --- |
| **Source** | **df** | **MS** | **MS** | **MS** | **MS** | **MS** | **MS** | **MS** | **MS** |
| **Main Effects** |  |  |  |  |  |  |  |  |  |
| **Stress** | 2 | 16090697*** | 28.32*** | 8.46*** | 5.03*** | 73.11*** | 41.55*** | 6.69*** | 31.57*** |
| **Treatments** | 2 | 2406807*** | 8.57*** | 2.09*** | 1.41*** | 1.66*** | 6.8** | 0.84** | 2.56*** |
| **Varieties** | 1 | 29246215*** | 5.73 *** | 2.86*** | 1.9** | 14.10*** | 3.01* | 5.98*** | 1.76*** |
| **Interaction** |  |  |  |  |  |  |  |  |  |
| **stress × Treatments** | 4 | 27703ns | 0.82*** | 0.46** | 0.30** | 2.12* | 2.19* | 0.69** | 0.43* |
| **stress × Varieties** | 2 | 313411ns | 0.56** | 0.57* | 0.33** | 2.60*** | 2.73*** | 0.98** | 0.51** |
| **Treatments × Varieties** | 2 | 13698ns | 0.43* | 0.51*** | 0.42*** | 2.45*** | 2.56** | 0.78*** | 0.60*** |
| **Stress × Treatments × Var** | 4 | 2840ns | 0.33* | 0.38* | 0.28* | 2.09* | 1.82* | 0.63** | 0.45* |
| **Error** | 36 | 96199 | 0.09 | 0.032 | 0.066 | 0.16 | 0.47 | 0.091 | 0.14 |
| **Total** | 53 |  |  |  |  |  |  |  |  |

|  |  | **Water Potential** | **Osmotic Potential** | **Turgor Pressure** | **Relative Water Content** | **Seed yield per plant** | **Harvest Index** | **Days to Maturity** | **Number of Pods Per Plant** |
| --- | --- | --- | --- | --- | --- | --- | --- | --- | --- |
| **Source** | **df** | **MS** | **MS** | **MS** | **MS** | **MS** | **MS** | **MS** | **MS** |
| **Main Effects** |  |  |  |  |  |  |  |  |  |
| **Stress** | 2 | 0.34* | 2.42*** | 1.46*** | 463*** | 16.90*** | 1488*** | 1826*** | 861*** |
| **Treatments** | 2 | 1.35*** | 0.72*** | 1.09** | 363*** | 5.84*** | 181** | 158*** | 146*** |
| **Varieties** | 1 | 0.54** | 0.18* | 1.05* | 312** | 8.10*** | 886*** | 105*** | 75*** |
| **Interaction** |  |  |  |  |  |  |  |  |  |
| **stress × Treatments** | 4 | 1.23*** | 0.32* | 1.06** | 0.30** | 1.12* | 119* | 21** | 14** |
| **stress × Varieties** | 2 | 0.93* | 0.56*** | 1.27*** | 0.24** | 1.64** | 273*** | 102*** | 270** |
| **Treatments × Varieties** | 2 | 0.89* | 0.38** | 0.81* | 0.12* | 1.97*** | 212** | 116*** | 11* |
| **Stress × Treatments × Var** | 4 | 1.19** | 0.48*** | 0.98* | 0.08* | 109* | 182* | 103*** | 13* |
| **Error** | 36 | 0.098 | 0.02 | 0.098 | 7.78 | 0.20 | 30.37 | 7.58 | 3.09 |
| **Total** | 53 |  |  |  |  |  |  |  |  |

**Table 5. ANOVA for evaluating the impact of cadmium stress on two mung bean cultivars NM-2011 and NM-2021 via zinc oxide nanoparticle application and their interactive effect on water relation and yield components**
